# The Effectiveness of Non-Pharmaceutical Interventions Upon Pregnancy-Related Low Back Pain: A Systematic Review and Meta-Analysis

**DOI:** 10.7759/cureus.13011

**Published:** 2021-01-30

**Authors:** Ioannis Koukoulithras, Alexandra Stamouli, Spyridon Kolokotsios, Minas Plexousakis, Christine Mavrogiannopoulou

**Affiliations:** 1 Department of Orthopaedic Surgery, University of Ioannina, Athens, GRC; 2 Department of Physical Therapy, University Hospital, University of West Attica, Athens, GRC

**Keywords:** pregnancy, low back pain, pain management, interventions, therapeutics, rehabilitation, systematic review and meta analysis

## Abstract

Introduction: Low back pain (LBP) is a very common pathology among pregnant women and various methods are used to reduce the pain. The aim of this study is to conduct an evidence-based systematic review and meta-analysis regarding the effectiveness of the interventions used to reduce low back pain related to pregnancy.

Methods and materials: The PEDro database, PubMed, and Cochrane Library were searched from January 2012 until December 2020 as well as the reference lists from identified articles. Studies of any non-pharmaceutical treatment to decrease low back pain were included but only randomized controlled trials were selected. The articles found were screened using the Preferred Reporting Items for Systematic Reviews and Meta-Analyses (PRISMA) question. Details about the type of intervention, sample size, outcome measures, results, and statistical significance were extracted from the selected studies. A meta-analysis for pain intensity was conducted and the I^2^ index as well as x^2 ^test were used to determine the heterogeneity between studies. A random-effects meta-analysis was carried out. The aim was to compare the effectiveness between various methods and the typical care provided on low back pain during pregnancy.

Results: From all the articles found in the mentioned databases only 13 studies met the criteria. In these studies, exercise, manipulation, ear acupuncture, Kinesio tape, transcutaneous electrical nerve stimulation (TENS), and neuroemotional technique were the interventions used. In the meta-analysis, six studies with 693 participants were included. The interventions were found to have in total a statistically significant effect on low back pain in comparison with the control group that included the typical care provided to pregnant women (95%CI: 0.08 (0.02,0.31), p<0,01) and they had a high heterogeneity (considerable, Tau² = 2.70; Chi² = 64.11, I² = 91%). Exercise and TENS were determined as more effective than the other types of interventions.

Conclusions: TENS and progressive muscle relaxation exercises accompanied by music were found to be the most effective interventions. Although exercise decreased LBP it was not found to have a statistically significant result even though it seems to improve the disability and quality of life of pregnant women. Osteopathic manual treatment (OMT), Kinesio tape, and ear acupuncture affected the lumbar pain intensity but the difference compared to typical care or sham treatment was not statistically significant, while yoga did not improve pregnancy-related LBP. Further research is needed to determine the effectiveness of the interventions mentioned.

## Introduction

Low back pain (LBP) is a common pathology and affects both men and women of all ages. LBP is also very frequent amongst women during pregnancy and has a great impact on their daily lives [1]. Other neuromusculoskeletal problems that occur during pregnancy are pubic pain, hip pain, knee pain, leg cramps, carpal tunnel syndrome, and De Quervain’s tenosynovitis [2]. Most women experience at least one of these symptoms during pregnancy and approximately one-quarter of them have a temporary disability [3]. 

Since antiquity LBP during pregnancy was known as well as identified for many centuries and described by Hippocrates, Vesalius, Pinean, Hunter, Velpeau, and many other scientists [1]. The latest studies have shown that the prevalence of back pain during pregnancy is 57.3% [4]. Almost one-third of these women undergo severe back pain and the quality of their daily lives is affected [5]. Symptoms of LBP could start from early in pregnancy until giving birth, but usually, the pain becomes more severe during the third semester of pregnancy and is described as dull pain [4]. Pregnancy LBP is usually related to sleep disorders and may affect the activities of daily living or quality of life [6]. In most cases of LBP (75.78%), the pain does not radiate to other parts of the body.

The biomechanics of the body has been transformed due to the increasing weight and width of the body, in particular of the abdomen, and can raise the action force exerted to the back [5]. Alteration in hormone levels (relaxin, progesterone, and estrogen) [7] can also affect the ligaments and the muscles attached to the chest and abdomen, resulting in weakness. Ligaments get weaker and stretch in preparation for labor, causing pain at the low back and pelvic girdle; therefore, all these create pressure on the uterus and make it protrude forward and cause lordosis to the spine [8]. The extra weight can cause axial loading to the spine, pressing the intervertebral discs, so it will likely increase pain to the lower back [7]. The etiology causing LBP during pregnancy is multidimensional and many factors have been blamed for causing this condition, which is why it is so difficult to carry out the appropriate treatment for each case [9]. Many pregnant women during this period prefer to avoid pharmaceutical treatment because of the side effects and turn toward less invasive and unlikely to harm treatments. 

Pennick et al. (2013) conducted a systematic review of the interventions aiming to reduce or treat pelvic as well as LBP during pregnancy [10]. The present study was inspired by their review with the difference of focusing only on LBP in pregnant women. It aims to collect new data on this matter, examining in a systematic way the different non-pharmaceutical methods used to prevent or decrease LBP as well as their effectiveness in pain and disability among pregnant women conducted from 2012 until today.

## Materials and methods

This systematic review was developed in compliance with Preferred Reporting Items for Systematic Reviews and Meta-Analyses (PRISMA) guidelines by Moher et al. [11] and it is considered to be an update of a previous publication by Liddle et al. [12] but with the difference of focusing only on LBP related to pregnancy rather than the lumbopelvic pain studied in that systematic review.

Search methods

The search was conducted through PubMed, PEDro, and Cochrane Library databases as well as the reference lists from identified articles. Only randomised controlled trials (RCTs) published within a nine-year period, from January 2012 until December 2020, were included. The search strategy was performed with the use of the following Medical Search Heading (MeSH) terms: "low back pain" AND "pregnancy" AND " therapeutics".

Study selection

The selection of the studies was based on the Population Intervention Comparison Outcome (PICO) question (Table 1). The primary screening of the potential articles found in the previously mentioned databases was carried out by three of the authors based on the title and the abstract. Each reviewer searched all databases independently and then they compared the outcomes and discussed any disagreement with a fourth author. The studies included were only RCTs because they as well as the analysis of quantitatively synthesized RCT data are considered as the gold standard for evaluating efficacy in clinical research and constitute evidence for medical treatment. Thus, RCT data are guiding physicians toward evidence-based therapy [13]. 

**Table 1:** PICO Question

P	Population/Problem/Patient	Pregnant women
I	Intervention	Non-pharmaceutical interventions
C	Comparison	Typical care
O	Outcome of interest	Reduce of pregnancy related Low Back Pain

Exclusion criteria

The exclusion criteria were determined at the beginning of the search. Articles with a publication date before 2012 were not included as well as the ones written in other languages than English or Greek. Studies where neither the abstract nor the full text was not available were also excluded. A large number of the articles found referred to LBP after or during the delivery of the embryo or they explored the effect of the interventions on both low back and pelvic pain. They were also excluded.

Methodological quality 

The assessment of the methodological quality of selected articles was conducted by four of the authors independently and was based on the criteria of the Physiotherapy Evidence Database (PEDro) scale [14] and Critical Appraisal Skills Programme (CASP) scale. The PEDro scale comprises 11 criteria, 10 of which evaluate the internal validity (criteria 2-9) and the sufficiency of statistical information of the study (criteria 10, 11) while the CASP scale consists of 11 questions but it examines the methodological quality and the value of the outcomes to the population. In the PEDro scale, articles with a score lower than 3/10 were categorized as «low» methodological quality, 4-6/10 as «moderate» and 7-10/10 as «high». In both scales if the answer in the first question (PEDro) or section (CASP) was negative the study was excluded from the meta-analysis. Also, all articles were examined and those with scores greater than 9 out of a score of 16 (CASP) and 6 out of 10 (PEDro) were determined as acceptable quality. Τhe reviewers compared the results of their findings and qualitative assessment scoring of each article and any disagreement was resolved through discussion with the fifth author.

Data extraction

Data from each included article were extracted by each individual based on the following categories: interventions, sample size/group size, duration, follow-up, outcome measures, outcomes and statistical significance.

Meta-analysis

The meta-analysis aimed to determine the effectiveness of various treatments on low back pain related to pregnancy in comparison with the Usual Obstetric Care (UOBC). Statistical analysis and meta-analysis were conducted using Review Manager 5.4 software (Cochrane Collaboration, Copenhagen). The level of statistical significance was set at p<0.05 and the odds ratios (OR) were calculated for all dichotomous outcomes. Analyses for the RCTs were conducted using a random-effects model to evaluate whether the effect of LBP was different when examining evidence from interventions versus UOBC. 

For the RCTs that a meta-analysis was not possible, the results were presented as a narrative synthesis. In these studies, the data was incomplete (e.g., number of cases/controls not provided). The outcome of the meta-analysis was the number of women reporting pain after the intervention that they had received on the Visual Analogue Scale (VAS) or Roland-Morris Disability Questionnaire (RMDQ). Heterogeneity was quantified using the x^2^ test for heterogeneity and the I^2^ statistic [15]. I^2^ estimates the proportion of total variability between studies due to heterogeneity rather than chance alone. We considered I^2^ less than 25% to indicate low heterogeneity and I^2^ greater than 75% to indicate considerable heterogeneity [15].

## Results

Literature review results 

**Figure 1:**
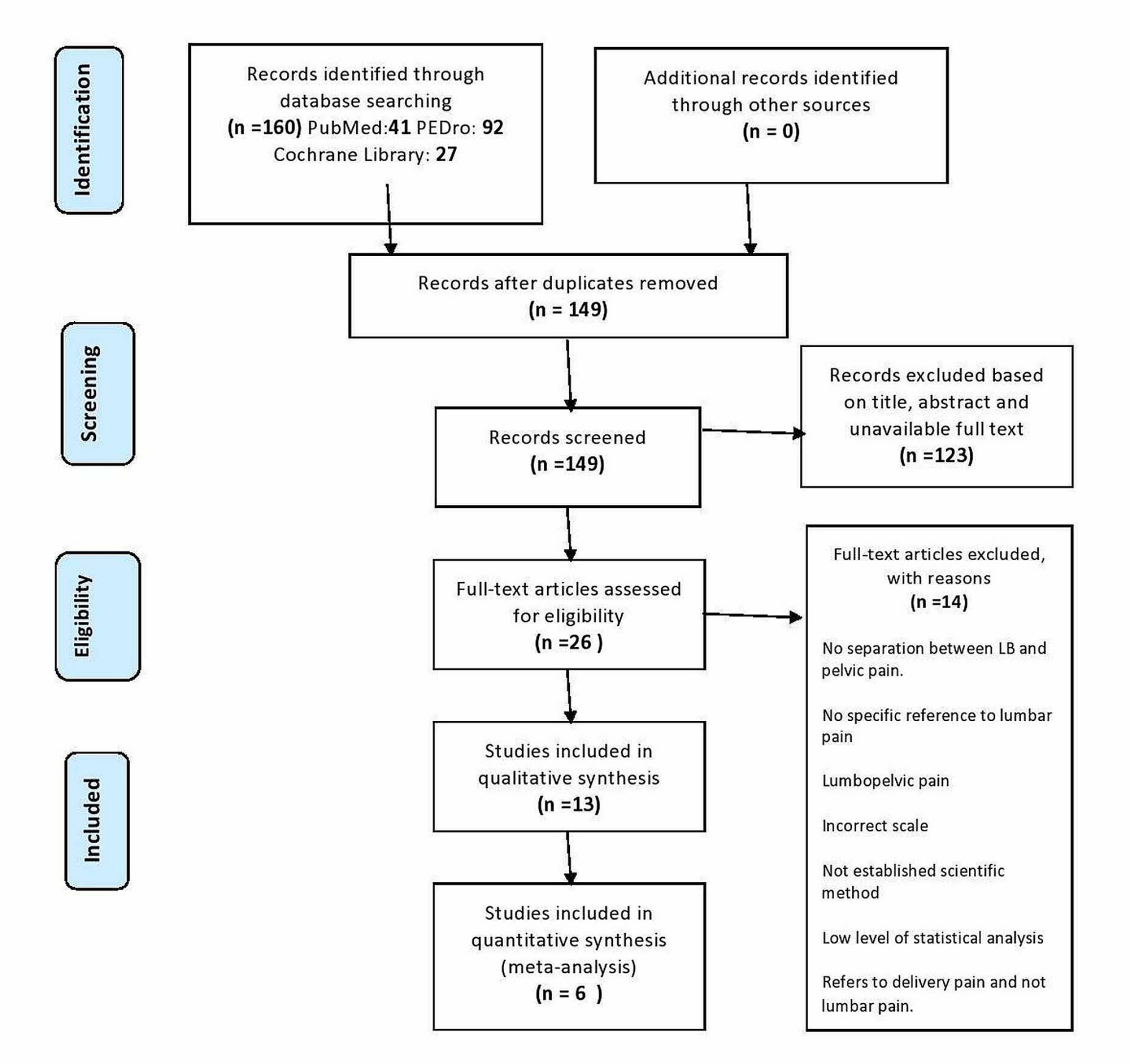
Preferred Reporting Items for Systematic Reviews and Meta-Analyses (PRISMA) Flow Diagram

A flow diagram outlining the systematic review process is provided (Figure 1). From the initial database search, 160 articles were collected (PubMed: 41; PEDro: 92; Cochrane Library: 27). Articles were reduced to 26 following the removal of duplicates and the application of the established exclusion criteria after their assessment. Based on the mentioned above inclusion and exclusion criteria and the design employed for the present systematic review, 13 RCTs were included for qualitative synthesis and 2213 patients were examined. After RCTs were identified and excluded with reasons (the search conducted included only articles between 2012 and 2020; so, a 2004 article is not suitable, no separation between LBP and pelvic pain, referring to pain generally, incorrect scale, not established scientific method, low level of statistical analysis, referring to delivery pain and not lumbar pain), they were reduced to 13. Information about the included articles is available in Table 2. Of the remaining 13 articles, seven did not meet the inclusion criteria for the meta-analysis. In the excluded articles there was not given the specific number of people who had the same or more pain after the intervention in the intervention group or the control group, and as intervention in the control group was not given UOBC. Therefore, six articles were available for meta-analysis. 

**Table 2:** Characteristics of articles LBP: Low Back Pain, RMDQ: Roland-Morris Disability Questionnaire, ΕQ-5D: EuroQol Questionnairre, VAS: Visual Analogue Score, UOBC: Usual Obstetric Care, OMT: Osteopathetic Manipulation, SUT: Sham Ultrasound Treatment, ODI: Oswestry Disability Index.

	Authors	Sample size	Intervention	Duration	Follow up	Outcome measures	Outcomes	Statistical significance
1	Backhausen, M. G. et al. (2017) [16]	516 Control Group (n=258) Intervention Group (n =258 )	water exercise vs standard prenatal care	October 2013 until May 2015	32 weeks	Low Back Pain Rating scale RMDQ EQ-5D and EQ-VAS	Low back pain intensity was significantly lower in the water exercise group vs the control group . No difference was found in the number of days spent on sick leave, disability due to low back pain nor self-rated general health.	Exercise group: 2.01 (95% CI 1.75–2.26) vs. 2.38 in the control group (95% CI 2.12–2.64) Mean difference = 0.38, 95% CI 0.02–0.74 p = 0.04
2	Haakstad, L. A. H. and Bø, K. et al. (2015) [17]	105 Exercise group (n=52) Control group (n=53)	Exercise program vs Usual prenatal care	February to May (2008)	The first visit was between 12 and 24 weeks The second visit was between 36–38 weeks (after the intervention) The third visit was between 6–8 weeks after delivery (postpartum visit)	Questions concerning pregnancy complaints and Questions concerning the disability or severity of PGP/LBP	There were no statistically significant differences between the exercisers and controls in numbers reporting the 2 conditions after the intervention	Low back pain: OR=1.10, CI=0.47–2.60
3	Ozdemir, S. et al. (2015) [18]	96 Control Group (n = 48) Intervention Group (n = 48)	exercise program vs Usual care	December 2011–May 2012	First week Second week Third week Fourth week	VAS ODI	There was a statistically significant difference between the control and intervention groups with regards to the VAS scores.The final mean ODI2 scores for the control group were significantly higher than the mean ODI2 scores for the intervention group	p=0.001
4	M.H Eggen et.al. (2012) [19]	257 Intervention group (n=129) Control group (n=128)	Supervised exercise vs Standard care	March 2008 to June 2009	24, 28, 32, and 36 weeks	Self reported LBP Pain intensity in the morning and evening, disability 8-Item Short-Form Health Survey (SF-8) Physical Component Summary (PCS) Mental Component Summary (MCS) scores	Supervised group exercise did not reduce the prevalence of LBP.	Prevalence of LBP Odds Ratio=0.77, 95% CI=0.50 to 1.19 Secondary outcomes -0.4 (95% CI=-0.8 to 0.1) for pain intensity in the morning, -0.4 (95% CI=-1.0 to 0.2) for pain intensity in the evening, -1.0 (95% CI=-2.2 to 0.0) for disability, 1.8 (95% CI=0.0 to 3.7) for the SF-8 PCS, and -0.6 (95% CI=-2.2 to 1.4) for the SF-8 MCS.
5	Peterson et.al. (2012) [20]	57 Exercise (n=22) SMT (n=15) NET (n=20)	Exercise VSNeuro Emotional Technique (NET) Exercise VS Spinal manipulation (SMT) NET VS SMT	August 2009 to April 2011	once monthly until 28 weeks gestation, twice monthly until 36 weeks gestation, and weekly thereafter	Roland Morris Disability Questionnaire Numeric Pain Rating Scale.	SMT and exercise generally performed slightly better than did NET for improving function and decreasing pain, but the study was not powered to detect the between-group differences as statistically significant.	Exercise vs NET −0.3 (−3.7, 3.0) P value =0.712 Exercise vs SMT −2.0 (−5.6, 1.6) P Value= 0.995 SMT vs NET 1.6 (−1.5, 4.8) P Value= 0.453
6	Mirmolaei, S. T. et al. (2018) [21]	180 Control Group (n = 90) Intervention Group (n = 90)	Physical training program vs Prenatal care	2010-2011	12-week	VAS ODI	Pain and physical disability decreased significantly in the intervention group	P<0.05
7	Akmeşe et al. (2014) [22]	66 Control Group (n = 33) Intervention GroupI (n = 33)	Progressive muscle relaxation exercises accompanied by Music	8 weeks	4 weeks 8 weeks	VAS SF-36 Personal information form	No significant differences in sociodemographic or obstetric characteristics were observed between the groups VAS Control Before treatment: 7.69 After 4 weeks: 8.42 After 8 weeks: 9.03 PMR Group Before treatment: 7.78 After 4 weeks: 5.21 After 8 weeks: 3.72 SF-36 Subscale Scores Significant differences were found for every subscale at 4 and 8 weeks respectively	P <0.001 P <0.001
8	Keskin et al. (2012) [23]	79 control group (n=21) exercise (n=19) acetaminophen (n=19) TENS (n=20)	Exercise vs Acetaminophen vs TENS vs Typical care	3 weeks	At the end of the trial	VAS RMDQ	Control Before treatment: 6 After treatment: 7 Exercise Before treatment: 7 After treatment: 6 Acetaminophen Before treatment: 6 After treatment: 5 TENS Before treatment: 7 After treatment: 4	control Difference : 1 p (Z)b: 0.003 (2.952) Exercise Difference : -1 p (Z)b: <0.001 (3.804) Acetaminophen Difference : -1 p (Z)b: <0.001 (3.946) TENS Difference : -4 p (Z)b: <0.001 (4.005)
9	Licciardone JC et.al. (2013) [24]	146 UOBC + OMT (n=49) UOBC +SUT (n=48) UOBC (n=49)	UOBC + OMT VS UOBC + SUT VS UOBC	2003 to 2006	Obstetric visits weeks 30, 32, 34, 36, 37, 38, and 39.	RMDQs	Patients who received UOBC + OMT were significantly less likely to experience progressive Back Specific Dysfunction.	UOBC +OMT VS UOBC + SUT (RR, 0.6; 95% CI, 0.3-1.0; P=0.046) UOBC +OMT VS UOBC (RR, 0.4; 95% CI, 0.2-0.7; P<0.001
10	Hensel et al. (2015) [25]	400 typical care (n=133) typical care + OMT (n=136) typical care+placebo ultrasound(n=131)	usual care (UCO) vs usual care+OMT (OMT) vs usual care+ placebo ultrasound (PUT)	9 weeks	at the end of trial	pain back related functioning QVAS RMDQ	OMT and PUT had significant difference compared to UCO not significant difference between OMT and PUT	OMT vs PUT RMDQ 95%CI: 0.21(-0.73-1.14) p>.999 CPI: 95%CI: .15(-3.07-3.36) p>.999 OMT vs UCO RMDQ 95%CI: -2.25(-3.18- -1.32) p<0.001 p="" cpi:=""
11	Kaplan S. et al., (2016) [26]	71 Intervention group(n=36) Control group (n=35)	Intervention group was treated with paracetamol plus Kinesio taping Control group received only paracetamol	July 27, 2015 to December 1, 2015.	5th day	Visual analogue scale (VAS) the scores of the Turkish version of the RolandMorris Disability Questionnaire (RMDQ)	In both groups pain intensity during rest, pain intensity during movement, and Roland-Morris Disability Questionnaire were significantly reduced at day 5 compared with baseline Τhe Kinesio tape group was significantly superior than the control group in all the outcome measures considering the change data from baseline to day 5	Intervention group vs Control group VAS (rest)= 0.357 p<0.001 VAS (motion)= 0.590 p<0.001 RMDQ= 0.085 p<0.001
12	Holden S et.al. (2019) [27]	20 Yoga (n=11) Control (n=9)	Prenatal Yoga VS educational attention control	April 2015 to December 2015	every 4 weeks, and 6 weeks postpartum.	Roland Morris Disability Questionnaire visual analogue scale(VAS)	No differences in back pain were observed between 2 groups.	8 weeks effect RMDQ 0.004 (1.98) p = 0.99 12 weeks effect RMDQ 0.82 (1.98) p=0.68
13	Vas J. et al., (2019) [28]	220 verum ear acupuncture (VEAc) (n=55) nonspecific ear acupuncture (NSEAc) (n=55) placebo ear acupuncture (PEAc) (n=55), or no acupuncture (SOC) (n=55)	Verum ear acupuncture (VEAc) vs Nonspecific ear acupuncture (NSEAc) vs Placebo ear acupuncture (PEAc) vs No acupuncture (SOC)	March 2014 to December 2016	1 week after starting treatment (T1), 2 weeks after treatment (T2), 3 months postpartum (T3), 1 year after study enrollment (T4)	0 to 100 mm VAS Roland-Morris Disability Questionnaire (RMDQ) Short Form-12 Health Survey (SF-12)	Primary outcome: VEAc group greater reduction in pain intensity then SOC group at T2 and T3 No statistically significant differences in pain score reductions were found between the NSEAc or PEAc group and the SOC group Secondary outcomes: VEAc group better results in RMDQ scores (indicating less disability) at T2 compared with the SOC group The VEAc group had batter SF-12 scores	T2: VEAc vs SOC CI: 15.3-34.9 vs. 65.8%, 95% CI: 56.2- 75.3 T3: VEAc vs SOC CI: 55.3-80.5 vs. 93.8%, 95% CI: 88.7-99.0 Secondary outcomes: no information available

Methodological assessment of the sample according to the PEDro and CASP Scale

All the studies included in the sample were assessed and rated using the criteria of the PEDro (Table 3) and CASP Scale (Table 4) and then were classified into groups determined as «High», «Moderate» and «Low» methodological quality. Specifically, 13 studies were analyzed in which six of them were evaluated as of high (7-10/10), seven as moderate (5-6/10) and none of the included RCTs as low methodological quality. 

**Table 3:** Physiotherapy Evidence Database (PEDro) Scale

	Eligibility criteria	Random allocation	Concealed allocation	Baseline comparability	Blind subjects	Blind therapists	Blind assessors	Adequate follow-up	Intention-to-treat analysis	Between-group comparisons	Point estimates and variability	/10
Backhausen et al. (2017) [16]	YES	YES	YES	YES	NO	NO	NO	YES	YES	YES	YES	7
Haakstad et al. (2015) [17]	YES	YES	NO	YES	NO	NO	YES	YES	YES	YES	YES	7
Ozdemir et al. (2015) [18]	YES	YES	YES	YES	NO	NO	NO	YES	YES	YES	YES	7
													Eggen et al. (2012) [19]	YES	YES	YES	YES	NO	NO	YES	YES	NO	YES	YES	7
Caroline D Peterson et.al. (2012) [20]	YES	YES	YES	YES	NO	NO	NO	YES	YES	YES	YES	7
Mirmolaei et al. (2018) [21]	YES	YES	NO	YES	NO	NO	NO	YES	NO	YES	YES	5
Akmeşe et al. (2014) [22]	YES	YES	NO	YES	NO	NO	NO	YES	NO	YES	YES	5
Keskin et al. (2012) [23]	YES	YES	YES	YES	NO	NO	NO	YES	NO	YES	YES	6
Licciardone et.al. (2013) [24]	YES	YES	NO	YES	NO	NO	YES	YES	YES	YES	YES	7
Hensel et al. (2015) [25]	YES	YES	YES	YES	NO	NO	NO	NO	YES	YES	YES	6
Kaplan et al. (2016) [26]	YES	YES	NO	YES	NO	NO	YES	YES	NO	YES	YES	6
Holden S. et.al (2019) [27]	YES	YES	NO	YES	NO	NO	NO	NO	YES	YES	YES	5
Jorge vas et al.(2019) [28]	YES	YES	NO	YES	NO	NO	NO	YES	YES	YES	YES	6

**Table 4:** Critical Appraisal Skills Programme (CASP) Scale

	A: Validity			B: Methodological validity			C: Results			D: Help locally	
	Did the study address a clearly focused research question?	Was the assignment of participants to interventions randomised?	Were all participants who entered the study accounted for at its conclusion?	Were the participants ‘blind’ to intervention they were given? Were the investigators ‘blind’ to the intervention they were giving to participants? Were the people assessing /analysing outcome/s ‘blinded’?	Were the study groups similar at the start of the randomised controlled trial?	Apart from the experimental intervention, did each study group receive the same level of care (that is, were they treated equally)?	Were the effects of intervention reported comprehensively?	Was the precision of the estimate of the intervention or treatment effect reported?	Do the benefits of the experimental intervention outweigh the harms and costs?	Can the results be applied to your local population/in your context?	Would the experimental intervention provide greater value to the people in your care than any of the existing interventions?
Backhausen et al. (2017) [16]	YES	YES	NO	NO	YES	YES	YES	YES	CANT TELL	YES	YES
Haakstad et al. (2015) [17]	YES	YES	NO	NO	YES	YES	YES	YES	CAN’T TELL	YES	YES
Ozdemir et al. (2015) [18]	YES	YES	YES	NO	YES	YES	YES	YES	YES	YES	YES
EGGEN ET AL. (2012) [19]	YES	YES	NO	NO	YES	YES	YES	YES	CAN’T TELL	YES	YES
Peterson CD et al. (2012) [20]	YES	YES	NO	NO	NO	NO	YES	YES	CAN’T TELL	YES	YES
Mirmolaei et al. (2018) [21]	YES	YES	NO	NO	YES	YES	YES	YES	CAN’T TELL	YES	YES
Akmeşe et al. (2014) [22]	YES	YES	NO	NO	YES	NO	CAN'T TELL	NO	YES	YES	YES
Keskin et al. (2012) [23]	YES	YES	NO	CAN’T TELL	YES	NO	CAN’T TELL	NO	YES	YES	YES
Licciardone et al. (2013) [24]	YES	YES	NO	NO	YES	YES	YES	YES	CAN’T TELL	YES	YES
Hensel et al. (2015) [25]	YES	YES	NO	YES	YES	YES	YES	YES	YES	YES	YES
Kaplan et al. (2016) [26]	YES	YES	NO	NO	YES	YES	YES	NO	YES	YES	CAN’T TELL
Holden S et.al .(2019) [27]	YES	YES	NO	NO	YES	YES	YES	YES	CAN’T TELL	YES	YES
Jorge vas et al. (2019) [28]	YES	YES	NO	NO	YES	YES	CAN’T TELL	YES	CAN’T TELL	YES	YES

Specifically, eight RCTs used exercise as the main intervention, five of them were evaluated as of high methodological quality (Backhausen et al. [16], Haakstad et al. [17], Ozdemir et al. [18], Eggen et al. [19], Peterson et al. [20]) and three of them as of moderate methodological quality (Mirmolaei et al. [21], Akmese et al. [22], Keskin et al. [23]). Three RCTs compared multiple interventions and only one of them was evaluated as of high methodological quality (Licciardone et al. [24]) while the other two were evaluated as moderate (Keskin et al. [23], Hensel et al. [25]). Lastly, only one RCT for each of the following interventions was found: Kinesio tape (Kaplan et al. [26]), yoga (Holden et al. [27]), ear acupuncture (Vas et al. [28]) and all of them were evaluated as moderate methodological quality.

The CASP Randomised Controlled Trial Standard Checklist was used to evaluate the RCTs in addition to the PEDro scale. The CASP Scale helped to develop an evidence-based approach while aiming to assist individuals to develop skills in order to understand better the research evidence and help them apply evidence in clinical practice. Although, the CASP Randomised Controlled Trial Standard Checklist was used to further evaluate the quality of RCTs. Although there is a potential risk of bias. In addition, it is not recommended to use a scoring system with this tool. 

Meta-analysis

**Figure 2:**
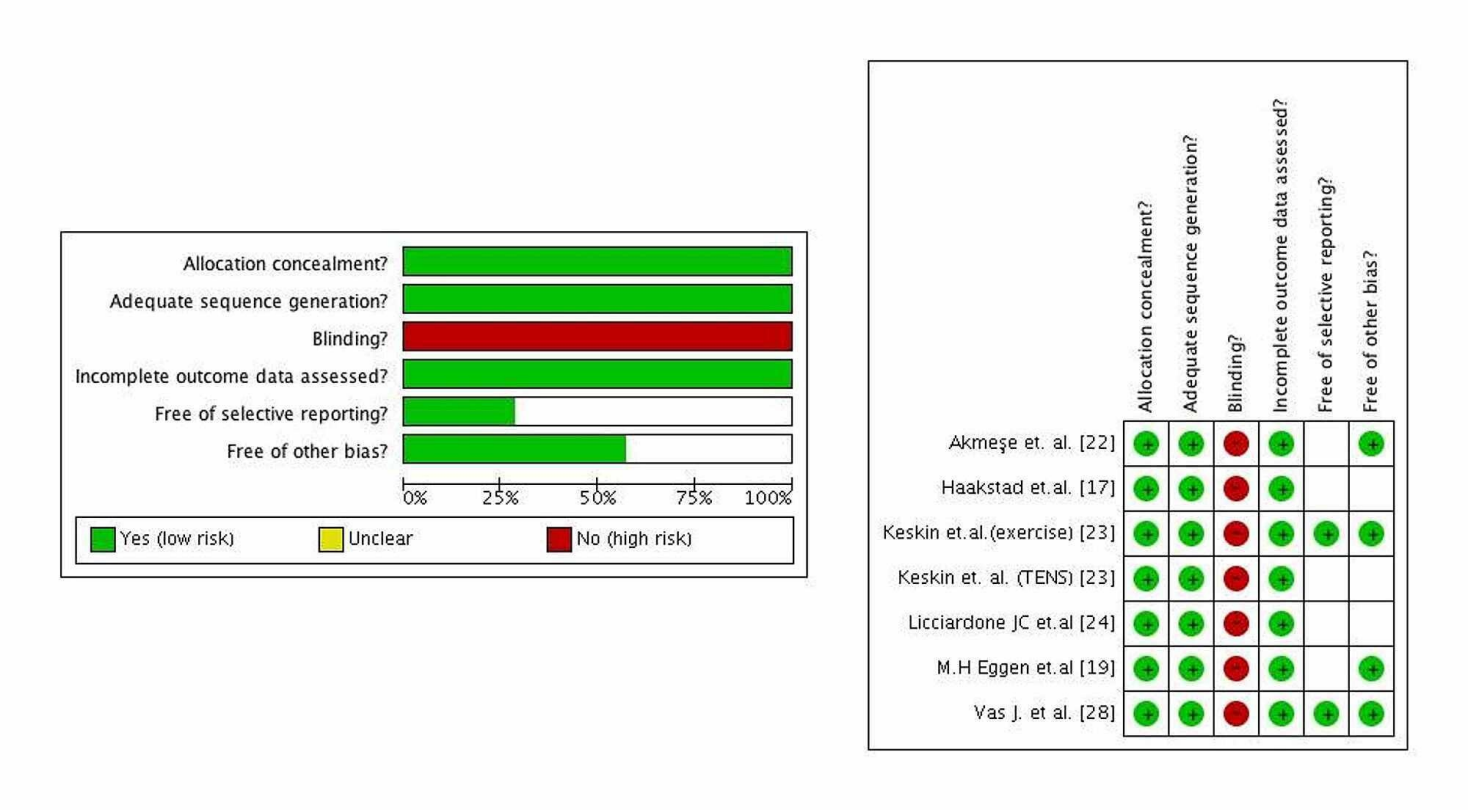
Risk-of-bias assessment of randomized controlled trials included in the meta-analysis.

Overall, study quality was high. There was ‘high’ quality evidence from the six RCTs (n=693) regarding the association between intervention and odds of LBP (Figure 2). There was no serious risk of bias, indirectness of the interventions, and imprecision (Figure 2). A summary of the findings with respect to outcomes is presented along with an evaluation of the quality of the evidence based on the grading of recommendations assessment, development and evaluation (GRADE) [29].

The study had a high heterogeneity (considerable, Tau² = 2.70; Chi² = 64.11, I² = 91%). The heterogeneity was neither statistical nor methodological. It was clinical due to the high differences of participants in the studies and the high effectiveness of the TENS [23], exercise [23], and progressive muscle relaxation exercises accompanied by music interventions [22].

**Figure 3:**
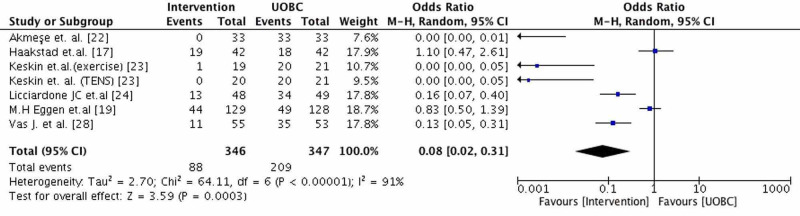
Effects of intervention compared with typical care (UOBC) on low back pain (LBP). Outcome: Number of women with LBP after the last intervention Note: CI, confidence interval; df, degrees of freedom; M-H, Mantel-Haenszel method; UOBC, Usual Obstetric Care

At the meta-analysis (Figure 3), six RCTs were included and the total number of pregnant women participating was 693 (intervention and control groups). Although, seven interventions were studied because in one article (Keskin et al. [23]) two different methods with two different groups were compared to typical care (TENS and exercise). Studies whose data were incomplete were excluded. Seven RCTs (Backhausen et al. [16], Ozdemir et al. [18], Peterson et al. [20], Mirmolaei et al. [21], Hensel et al. [25], Kaplan et al. [26], Holden et al. [27]) did not report the number of pregnant women with pain after the last intervention in both groups. These studies reported the mean VAS score or mean RMDQ score. Also, in some studies the control group didn’t take UOBC. The effectiveness of interventions versus typical care was evaluated in pregnant women with LBP and the outcome was the number of women reporting LBP after the last intervention. For the intervention and control group were included only women whose pain was not reduced or was at the same level after the last intervention. The mean week of gestation of the pregnant women who took part in studies was 24. The interventions found in the included articles in the meta-analysis were exercise, OMT, ear acupuncture, TENS, and progressive muscle relaxation exercises accompanied by music. 

The pooled estimate for the interventions versus UOBC was statistically significant (p=0.0003). The interventions are very effective for the reduction of LBP during pregnancy 95%CI: 0.08 [0.02-0.31] (Figure 3). Also, it was found that exercise (Keskin et al. [23] 95%CI: [0.00-0.05]), muscle relaxation exercises accompanied by music (Akmese et al. [22] 95%CI: [0.00-0.01]) and TENS (Keskin et al. [23] 95%CI: [0.00-0.05]) are more effective than the other types of interventions respectively. The heterogeneity was high due to the high differences of participants in the studies and the high effectiveness of the three interventions discussed previously. The results from the studies of Eggen et al. [19] and Haakstad et al. [17] weren’t statistically significant and there was no reduction in LBP. Although, in these studies there were statistically significant results in the secondary outcomes such as disability and quality of life. Last but not least, OMT (Licciardone et al. [24]) and ear acupuncture (Vas et al. [28]) seem to have statistically significant results in the reduction of LBP (95%CI 0.16 [0.07,0.40]), 95%CI [0.05,0.31], respectively).

## Discussion

LBP is a common pathology among pregnant women related to the musculoskeletal, neurological and consequently posture changes that occur in their bodies and was found to affect the quality of their lives and increase their disability. Relapse rates are high in subsequent pregnancies, and a postpartum prevalence of 24.7% (range 0.6% to 67%) underlines the importance of developing effective treatment programs for this condition. Despite these figures, it is estimated that over 50% of women receive little or no intervention from healthcare providers [12]. The aim of the study was to add the newest data upon this pathology to the previous systematic review and meta-analysis conducted by Liddle et al. (2015) [12] and to examine the effectiveness of the existing interventions used to decrease LBP.

In total, 160 articles were found in the three databases searched, but only 13 of them with 2213 pregnant women participating and six different interventions examined were included in the systematic review. Eight articles were about exercise, two about OMT, and one of each of the following interventions: TENS, Kinesio tape, yoga, and ear acupuncture. From them, only six studies (n=693) meet the criteria for the meta-analysis and of them four examined the effect of exercise, one of TENS, one of OMT, and one of ear acupuncture.

Exercise was found to be a very common practice used to decrease LBP and the majority of the relative studies were categorized as of «high methodological quality» (Tables 3, 4). The results from the meta-analysis showed that exercise had a statistically significant effect (Keskin et al. [23]: 95%CI: [0.00-0.05], Akmese et al. [22]: 95%CI: [0.00-0.01]) compared with typical care. Akmese et al. in their research examined the effect of exercise combined with music on LBP. In the studies of Haakstad et al. [17] and Eggen et al. [19] it was not found effective upon the intensity of pain in comparison with UOBC, although they have shown a great effect on disability and quality of life of the participating pregnant women. From the articles that were excluded from the meta-analysis, the RCTs of Backhausen et al. [16] (n=516), Peterson et al. [20], and Mirmolaei et al. [21] seem to reduce the pain intensity but they do not appear to have a statistical significance. The results of this study regarding exercise are in agreement with them from the systematic review and meta-analysis of Davenport et al. [8].

The exercise components of the interventions included strength and stretching (Haakstad et al. [17]), endurance training (Haakstad et al. [17]), pelvic tilt exercises, stretching and mild isometric abdominal contractions (Keskin et al. [23]), progressive muscle relaxation exercises accompanied by music (Akmese et al. [22]) as well as aerobic and stretching exercises (Eggen et al. [19]). The quality of the evidence was high. The women who received typical care had regular visits at the maternity primary care centres, information and advice for health complaints and explanations of the causes of LBP.

The high quality of evidence from the meta-analysis has shown that OMT if received in addition to usual care, it has a greater impact on the decrease of LBP and functional disability than the usual care provided without OMT (Licciardone et.al [24]: 95%CI 0.16 [0.07,0.40]). This study has also been classified as of «high methodological quality» from PEDro scale (Table 3). A large study (n=400) of moderate quality that was excluded from the meta-analysis but included in the systematic review in which OMT was compared to usual care and placebo ultrasound, has found a greater decrease in LBP from OMT than UOBS or placebo ultrasound (PUT) and statistically important difference (Hensel et al. [25], p<0.001). So OMT was found to have a great effect on the pain intensity and functional status of pregnant women but more research is needed in order to create more high-quality articles, something that is also the conclusion of the systematic review and meta-analysis conducted by Franke et al. [30]. 

Another intervention examined was ear acupuncture, specifically Verum ear acupuncture (VEAc), nonspecific ear acupuncture (NSEAc), and placebo ear acupuncture (PEAc) in comparison to typical care, a single study carried out by Vas et al. [28]. The meta-analysis showed that ear acupuncture had statistically significant results (95%CI [0.05,0.31]) and the lumbar pain of the participating pregnant women was decreased. TENS was also found to be very effective upon pain intensity and functional disability during pregnancy more than acetaminophen and had a significant statistical difference (95%CI: [0.00-0.05]) but the study of Keskin et al. [23] was of «moderate quality» based on the PEDro scale (Tables 3, 4).

The following studies were from the ones included in the systematic analysis but excluded from the meta-analysis. Kinesio tape additional to paracetamol was found to have better results on LBP compared to only paracetamol in a study conducted by Kaplan et al. [26]. The research showed that the intervention was statistically important (p<0.001), but further study is required in order to determine that effect. Furthermore, Holden et al. [27] in a moderate quality study examined yoga in comparison to educational attention and no difference was found.

The small number of related studies, as well as the small population of the participants in the majority of studies, resulted in limited conclusions from this review. In most of the articles included there was a long-term follow up so the results of this review will also represent the long-term effectiveness of the mentioned interventions. In addition, the high heterogeneity that was found was the result of the high difference of the participants in the studies as well as the high effectiveness of TENS, exercise, and muscle relaxation exercises accompanied by music and that consequently means that further research should be conducted in order to determine the effectiveness of the mentioned interventions on the LBP during pregnancy.

This study was considered to be the updated version of the systematic review and meta-analysis of Liddle et al. (2015) [12] and includes newer studies of high and moderate quality than they used in their research.

## Conclusions

LBP is a very common pathology that appears in all people but there is a very high prevalence of it during pregnancy so there is a need to determine effective interventions to reduce or better treat the problem better. In summary, TENS and muscle relaxation exercises accompanied by music were found to be the most effective interventions and they have a statistically important impact on lumbar pain. Various kinds of exercise were examined and they seem to reduce the pain intensity. Although most of the articles regarding exercise were of a high methodological standard, the results were not in total statistically significant but there was a great impact on the secondary outcomes such as disability and quality of life. Ear acupuncture and Kinesio tape, even though they were found effective in decreasing LBP, the results were not significant while yoga was neither effective nor statistically significant. Due to the high heterogeneity of the studies included in the meta-analysis the effectiveness of the described methods as well as the statistically significant interventions should be examined further in future studies.
